# Accurate Placement and Revisions for Cervical Pedicle Screws Placed With or Without Navigation: A Systematic Review and Meta-Analysis

**DOI:** 10.1177/21925682231196456

**Published:** 2023-08-19

**Authors:** B. J. J. Bindels, B. E. G. Dronkers, M. L. J. Smits, J. J. Verlaan

**Affiliations:** 1 Department of Orthopedic Surgery, 8124 University Medical Center Utrecht, Utrecht, The Netherlands; 2 Department of Radiology, 8124 University Medical Center Utrecht, Utrecht, The Netherlands

**Keywords:** surgical navigation, computer-assisted surgery, spine surgery, systematic review, accuracy, complications, pedicle screw, cervical spine

## Abstract

**Study Design:**

Systematic review and meta-analysis.

**Objectives:**

To evaluate the accuracy of placement for cervical pedicle screws with and without the use of spinal navigation.

**Methods:**

A structured search was conducted in electronic databases without any language or date restrictions. Eligible studies reported the proportion of accurately placed cervical pedicle screws measured on intraoperative or postoperative 3D imaging, and reported whether intraoperative navigation was used during screw placement. Randomized Studies (MINORS) criteria were used to evaluate the methodological quality of how accuracy was assessed for cervical pedicle screws.

**Results:**

After screening and critical appraisal, 4697 cervical pedicle screws from 18 studies were included in the meta-analysis. The pooled proportion for cervical pedicle screws with a breach up to 2 mm was 94% for navigated screws and did not differ from the pooled proportion for non-navigated screws (96%). The pooled proportion for cervical pedicle screws placed completely in the pedicle was 76% for navigated screws and did not differ from the pooled proportion for non-navigated screws (82%). Intraoperative screw reposition rates and screw revision rates as a result of postoperative imaging also did not differ between navigated and non-navigated screw placement.

**Conclusions:**

This systematic review and meta-analysis found that the use of spinal navigation systems does not significantly improve the accuracy of placement of cervical pedicle screws compared to screws placed without navigation. Future studies evaluating intraoperative navigation for cervical pedicle screw placement should focus on the learning curve, postoperative complications, and the complexity of surgical cases.

## Background

Placing pedicle screws in the cervical spine is technically challenging. The cervical pedicle is narrow and critical structures are close by such as the vertebral arteries, nerve roots, and spinal cord. The incidence of complications directly attributed to misplaced cervical pedicle screws varies significantly (.0%-5.7%), and reported rates of screw-related revision surgeries are low (1.0%-2.4%).^1,2^ However, complications caused by misplaced cervical pedicle screws may be severe and irreversible, or even lethal.^3-7^

Spinal navigation is an intraoperative guidance method developed to gain more control during interventions such as pedicle screw placement. Spinal navigation provides surgeons with continuous three-dimensional (3D) visual feedback on the position of a pedicle screw relative to the bony anatomy of the spine throughout the procedure. Without navigation, spine surgeons have to rely, apart from haptic feedback, on static intraoperative – or postoperative – imaging that only provides feedback on a screw’s position whenever an image is obtained.

Spinal navigation systems have shown potential to improve on the accuracy of placement of pedicle screws and reduce screw-related revision surgeries in the thoracic and lumbar spine.^8,9^ However, because acquiring and maintaining spinal navigation systems and their equipment is expensive, discussion remains if their costs outweigh the potential benefits.^10-12^ When used in high-risk surgical anatomy, including the cervical spine, even relatively small contributions in accuracy may justify the use of spinal navigation systems.

We conducted a systematic review and meta-analysis to evaluate the accuracy of placement for cervical pedicle screws placed with the help of navigation compared with screws placed without navigation.

## Methods

The present review adhered to guidelines of Meta-analysis Of Observational Studies in Epidemiology (MOOSE).^13^ The protocol for this review was published in the PROSPERO international prospective register of systematic reviews [CRD42022307501].^14^ This review required no approval from an international review board, and because no original patient data was used, obtaining informed consent did not apply to this study.

### Search Strategy

We conducted a structured search to identify all articles reporting on cervical pedicle screw placement using the electronic databases PubMed, Embase and Cochrane, without any language or date restrictions, on June 13, 2023. The following keywords and their synonyms were combined: “fluoroscopy or navigation or free-hand or robotic”, “pedicle screw” and “cervical spine” (Supplement 1).

### Selection Process

After duplicate removal using EndNote (The EndNote Team, Philadelphia, USA, version X9), title and abstract of all studies were independently assessed for eligibility by 2 authors (author 1 and author 2). If eligibility could not be determined based on title and abstract, the full text was reviewed. Any disagreements were resolved by consensus. We used Rayyan systematic review software for the screening of studies.^15^

Eligible studies evaluated the proportion of accurately placed pedicle screws in the cervical spine. Studies had to report what intraoperative imaging modality surgeons had used during the surgery and if they had used navigation equipment to place cervical pedicle screws. Accuracy of placement of pedicle screws had to be measured on an intraoperative or postoperative 3D image (e.g., computed tomography (CT) or cone-beam CT (CBCT)). Studies had to provide the proportion of cervical pedicle screws that breached the pedicle wall with more or less than 2 millimeters (mm) in any direction, or with more or less than 50% of the screw diameter in any direction as long as they used screws with a diameter of 3.5-4.0 mm. If a study did not report the screw diameter but assessed the accurate placement of pedicle screws based on the percentage of the screw diameter breaching the pedicle wall, the corresponding author of the pertaining study was contacted to provide screw diameters.

Only studies with more than ten patients were included. Studies that reported on other types of cervical screws, such as lamina or lateral mass screws, were included as long as they separately provided the accuracy of the cervical pedicle screws placed. If it was unclear how many patients underwent cervical pedicle screw fixation, we still included the study if we could extract the number and accuracy of the cervical pedicle screws placed.

No restrictions were applied regarding the technique for screw placement used (e.g., free-hand or robotic), the surgical approach (e.g., minimally invasive or open), or indication for surgical treatment. Exclusion criteria were conference abstracts, reviews, editorials, non-human or cadaveric studies, studies not written in English, French, German or Spanish, and studies for which a full text could not be retrieved.

### Data Extraction and Quality Assessment

Two authors (BJJB and BEGB) independently assessed the quality of each included study and extracted all data. Any discordances between reviewers were discussed with a third author (JJV) until consensus was achieved.

Data was collected for design and funding of the study, patient demographics, indication for surgery, surgical approach, cervical levels treated, method used to place pedicle screws, intraoperative imaging modalities and navigation system used, method used to assess accuracy of placement, and the accuracy of placement. Additionally, data was collected for the number of intraoperatively repositioned cervical pedicle screws and screws revised as a result of postoperative imaging.

Cervical pedicle screws were classified as navigated screws or non-navigated screws based on the intraoperative guidance method used for pedicle screw placement. Navigated screws were screws placed with the help of a navigational system that intraoperatively provided the surgeon with real-time 3D feedback of the screw position relative to the bony anatomy of the vertebra. Non-navigated screws were screws placed without the help of an intraoperative navigational system, with the surgeon just relying on visual/tactile feedback and/or static intraoperative imaging. If studies reported data for both navigated and non-navigated screws, the screws were divided into their respective group.

Cervical pedicle screws placed with a patient-specific pre-printed 3D guiding template for drilling or screwing were analyzed as a separate group and were not included in the primary analyses. When using a guiding template, surgeons rely less on the guidance from intraoperative imaging to place a pedicle screw, but they still receive essential patient-specific positional feedback from the template itself, making it a separate group not fitting our definition of navigated or non-navigated screw placement.

The primary outcome was the proportion of cervical pedicle screws placed completely in the pedicle or with a minor breach. A minor breach was defined as screws breaching the pedicle wall less than 2 mm or with less than 50% of the screw diameter in any direction. The 2 mm cut-off was chosen because breaches less than 2 mm are normally considered clinically irrelevant and breaches larger than 2 mm may cause clinical symptoms.^16-18^ If studies reported the accuracy of placement before and after intraoperative repositioning, we used the accuracy after intraoperative repositioning to allow for a valid comparison with studies only reporting the postoperative accuracy of placement. Secondary outcomes were the number of cervical pedicle screws placed completely within the pedicle, the number of screws with a major breach defined as screws breaching the pedicle more than 4 mm in any direction,^17^ the number of intraoperatively repositioned screws, and the number of screws revised as a result of postoperative imaging. All outcomes were assessed separately for navigated and non-navigated screws.

The Methodological Index for Non-Randomized Studies (MINORS) criteria were used to assess the methodological quality of included studies.^19^ The MINORS criteria comprise a 12-item checklist. Items are scored zero if the item is not reported, 1 if inadequately reported, or 2 if adequately reported. Comparative studies can score a maximum of 24 points, and non-comparative studies can score 16 points. Only studies comparing navigated cervical pedicle screw placement with non-navigated cervical pedicle screw placement were appraised as comparative studies, all other studies as non-comparative studies. We adjusted the MINORS criteria specifically for the primary outcome of the current review; the radiological accuracy of placement. Studies were only included in the meta-analysis if they included a consecutive group of patients, assessed accuracy of placement on intraoperative or postoperative CT or CBCT, and reported that the accuracy of placement was assessed by at least 1 independent observer. An independent observer was considered a person who was not involved in the surgeries (Supplement 2).

### Statistical Analysis

For navigated and non-navigated screws separately, the proportion of cervical pedicle screws with an insignificant breach was logit transformed for included studies. The logit-transformed proportions were pooled by conducting a meta-analysis via a generalized linear model using random effects (generalized linear mixed model). A generalized linear mixed model is preferred over classic meta-analyses for single proportions (e.g., arcsine or Freeman-Tukey double arcsine transformations) because it uses the exact binomial within-study distribution instead of a normal approximation. Additionally, a random-effect model better captures the uncertainty resulting from heterogeneity among studies than a fixed-effect model.^20^ The 2 pooled proportions were compared using a Wald-type test by fitting them into a fixed-effects meta-regression model. A fixed-effects model was applied because the generalized linear mixed models had already accounted for the (residual) heterogeneity.^21^ Pooled proportions were back-transformed and presented with a 95% confidence interval [95% CI]. Heterogeneity was assessed via the τ^2^, χ^2^, and I^2^ statistics. Pooling and subsequent comparison of secondary outcomes were performed using the same statistical methodology as for the primary outcome.

Potential publication bias was assessed by generating Doi plots using the Z-score on the vertical axis and the logit-transformed proportion on the horizontal axis. We used the LFK index to assess asymmetry in the Doi plots. The closer the value of the LFK index to zero, the more symmetrical the Doi plot is, and zero represents complete symmetry. LFK indices beyond ±1 were deemed consistent with asymmetry indicating publication bias. An LFK index >1 indicated positive publication bias, thus an overestimated accuracy of placement, and an index <1 indicated negative publication bias.^22,23^ All analyses were performed using R 4.0.3 software (The R Foundation for Statistical Computing, Vienna, Austria; ‘metafor’ and ‘metasens’ packages). *P*-values less than .05 denoted a statistically significant difference.

## Results

### Study Selection

**Figure 1.**
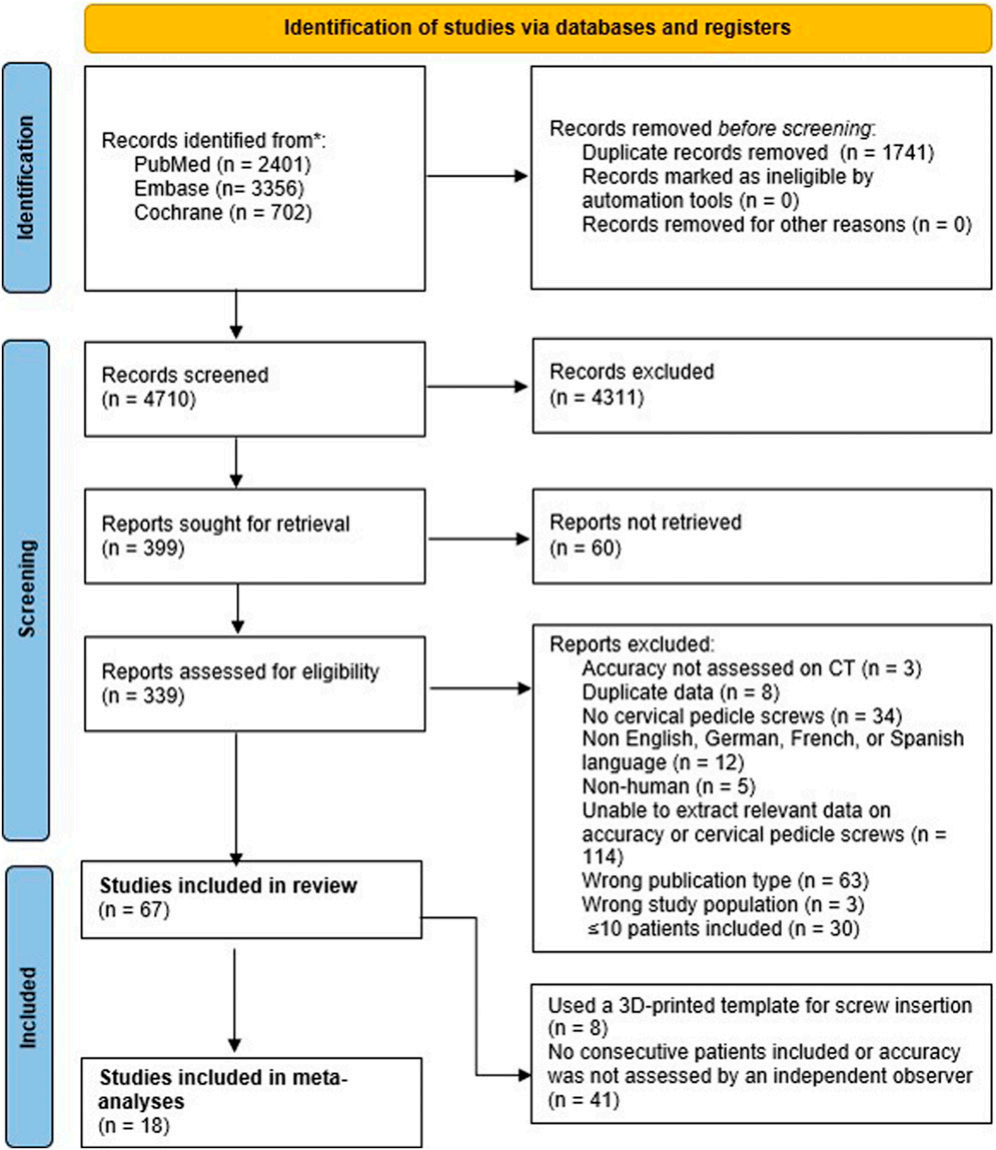
Flow diagram illustrating the searches, screening, and included number of studies.

The literature search identified 4710 unique studies. After title and abstract screening, 339 studies proceeded to full-text screening. Ultimately, 67 studies met the inclusion criteria (Figure 1).^18,24-89^

### Study Characteristics

All 67 included studies were non-randomized observational studies, of which 8 studies compared navigated screw placement to non-navigated screw placement. Surgical approach was open in 57 studies, minimally invasive or open in 7 studies, and not specified in 4 studies. In all 7 studies where patients were treated minimally invasively, surgeons used navigation for screw placement. In 56 studies, screws were placed with a free-hand technique, in ten studies with a 3D-printed guiding template, and in 3 studies a robotic arm was used (Table 1).

**Table 1.** Study Characteristics of All 67 Included Studies.

Author, year	Country	Funded	Study design	Comparing	Navigation vendor, system, type	Intraoperative imaging for navigation setup	Intraoperative imaging if no navigation	Screw insertion method	Surgical approach	Cervical levels	Patients	Surgical indication	CA
Studies assessing navigated and non-navigated screw placement													
Bertram, 2021	Germany	No	PCS	Nav vs non-nav	Brainlab, curve	iCT	2Dfluo	FH	Open	C2-C7	157	Multiple	20
Harel, 2019	Israel	No	RCS	Nav vs non-nav	MT, stealth station, NS	CBCT	2Dfluo	FH	Open	C2	14	Trauma	16
Inoue, 2022	Japan	No	RCS	Nav vs non-nav	MT, Stealth-Midas	CBCT	2Dfluo	FH	Open	C7	37^a^	Multiple	15
Lee J, 2020	South Korea	Yes	RCS	Nav vs non-nav	MT, stealth station, S7	CBCT	2Dfluo	FH	Open	C2	34	Multiple	19
Su, 2022	China	Yes	PCS	Nav vs non-nav	Tinavi, TiRobot	CBCT	2Dfluo	FH/robotic	Open	C1-C4	58	Multiple	20
Takamatsu, 2022	Japan	No	RCS	Nav vs non-nav	MT, stealth station, S8	iCT	2Dfluo	FH	Open	C2-C7	11	Trauma	15
Tanaka, 2021	Japan	No	RCS	Nav vs non-nav	MT, stealth station, S7	CBCT	2Dfluo	FH	MIS + open	C2-C7	25	Multiple	15
Zhou, 2023	China	Yes	RCS	Nav vs non-nav	Tinavi, TiRobot	CBCT	2Dfluo	FH/robotic	MIS + open	C1-C7	52	Trauma	20
Studies assessing navigated screw placement													
Barsa, 2016	Czech Republic	No	PCS	—	MT, stealth station, NS	iCT	—	FH	Open	C5-C7	18	Multiple	14
Bohoun, 2019	Japan	No	RCS	—	MT, stealth station, S7	CBCT	—	FH	NS	NS	12^a^	Multiple	10
Bredow, 2015	Germany	No	NS	—	Brainlab, VectorVision	CBCT	—	FH	Open	C2-C7	64	Multiple	12
Carl, 2019	Germany	Yes	RCS	Nav vs nav	Brainlab, curve	Preop CT + iCT	—	FH	Open	C2	16	Trauma	11
Chachan, 2018	Singapore	No	PCS	—	MT, stealth station, S7	iCT	—	FH	NS	C2-C7	44	Multiple	12
Gan, 2021	Singapore	No	RCS	—	MT, stealth station, S7	CBCT	—	FH	Open	C2-C7	82	Multiple	11
Habib, 2021	Switzerland	No	RCS	Nav vs nav	Brainlab, curve	iCT/CBCT	—	FH	Open	C3-C7	62	Multiple	11
Hecht, 2018	Germany	No	PCS	Nav vs nav	Brainlab, curve	iCT/CBCT	—	FH	Open	C2-C7	64^a^	Multiple	13
Hur, 2019	South Korea	No	RCS	—	MT, stealth station, S7	CBCT	—	FH	Open	C2	48	Multiple	12
Ito, 2008	Japan	No	PCS	—	MT, Stealth Station, NS	CBCT	—	FH	Open	C1-C7	50^a^	Multiple	11
Kim, 2014	South Korea	NR	RCS	—	MT, stealth station, NS Stryker, navigation, II	Preop CT + 2Dfluo	—	FH	Open	C2	18	Multiple	11
Kisinde, 2022	USA	No	RCS	—	MX, Stealth Edition	Preop CT + 2Dfluo	—	Robotic	NS	C2-C7	12	Multiple	10
Komatsubara, 2017	Japan	No	RCS	MIS vs open	MT, Stealth Station, NS	CBCT	—	FH	MIS + open	C2-C7	56	Trauma	10
Kumar, 2019	Singapore	No	RCS	—	MT, stealth station, S7	iCT	—	FH	Open	C2-C7	219^a^	Multiple	10
Lang, 2016	China	No	RCS	MIS vs open	NR	CBCT	—	FH	MIS + open	C2-C3	20	Trauma	9
Oikonomidis, 2020	Germany	No	RCS	—	NR	iCT	—	FH	Open	C3-C7	28	Multiple	10
Rajan, 2010	India	No	PCS	—	Brainlab, VectorVision	CBCT	—	FH	Open	NS	18	Multiple	12
Rienmuller, 2017	Austria	No	PCS	CPS vs TPS	Brainlab, curve	CBCT	—	FH	Open	C2-C7	107^a^	Multiple	14
Satake, 2021	Japan	No	RCS	Nav vs nav	MT, stealth station, S7	CBCT	—	FH	Open	C3-C6	47	Multiple	11
Scheufler, 2011	Austria	Yes	NS	—	Brainlab, NS	iCT	—	FH	MIS + open	C1-C7	27	NS	13
Shimokawa, 2017	Japan	NR	RCS	—	MT, stealth station, TREON	Preop CT + CBCT	—	FH	Open	C2-C7	128^a^	Multiple	10
Shin, 2022	Korea	No	RCS	—	MT, stealth station, S7	CBCT	—	FH	Open	C2-C7	51	Multiple	8
Sugimoto, 2010	Japan	NR	PCS	—	MT, stealth station, NS	CBCT	—	FH	Open	C2-C7	17	Multiple	9
Sugimoto, 2017	Japan	NR	NS	MIS vs open	MT, stealth station, NS	CBCT	—	FH	MIS + open	C2-C7	18	Tumor	10
Tauchi, 2013	Japan	No	RCS	—	Brainlab, VectorVision	iCT	—	FH	Open	C2-C7	46	Multiple	10
Tian, 2012	China	No	RCS	—	Stryker, navigation, NS	CBCT	—	FH	Open	C2-C3	14	Trauma	11
Tokioka, 2019	Japan	No	NS	MIS vs open	MT, stealth station, S7	CBCT	—	FH	MIS + open	C2-C6	86	Trauma	9
Wada, 2020	Japan	No	RCS	—	NR MT, Stealth-Midas	CBCT	—	Template	Open	C2-C7	64	Multiple	9
Zausinger, 2009	Germany	No	RCS	Nav vs nav	Brainlab, VectorVision	iCT	—	FH	NS	C1-C2	12	NS	10
Zhang, 2022	China	Yes	RCS	CPS vs LMS	MT, stealth station, NS	iCT	—	FH	Open	C3-C7	22	Trauma	10
Studies assessing non-navigated screw placement													
Cao, 2017	China	No	RCS	—	—	—	2Dfluo	FH	Open	C1-C2	87	Trauma	10
Farshad, 2022	Switzerland	No	RCS	—	—	—	None	Template	Open	C2-C7	12	Multiple	11
Hey, 2020	Singapore	No	PCS	CPS vs LMS	—	—	2Dfluo	FH	Open	C3-C7	21	Multiple	12
Hojo, 2014	Japan	No	RCS	—	—	—	2Dfluo	FH	Open	C2-C7	283	Multiple	12
Jiang, 2016	China	Yes	PCS	—	—	—	2Dfluo	Template	Open	C1-C2	32	Multiple	13
Kaneyama, 2015	Japan	NR	NS	—	—	—	None	Template	Open	C3-C6	20	Multiple	10
Kwon, 2022	Korea	Yes	PCS	—	—	—	2Dfluo	FH	Open	C3-C7	57	DG	13
Lee B, 2020	South Korea	No	RCS	Non-nav vs non-nav	—	—	None/2Dfluo	FH	Open	C1	25	Multiple	11
Lee, 2012	South Korea	No	RCS	—	—	—	2Dfluo	FH	Open	C3-C7	50	Multiple	12
Li, 2022	China	Yes	RCS	—	—	—	2Dfluo	FH	Open	C3-C7	32	Multiple	9
Liu, 2020	China	Yes	PCS	—	—	—	2Dfluo	FH	Open	C3-C7	32	Trauma	14
Lu, 2009	China	Yes	PCS	—	—	—	2Dfluo	Template	Open	C2-C7	25	Multiple	12
Mahesh, 2020	India	Yes	RCS	—	—	—	2Dfluo	FH	Open	C3-C7	99	Multiple	11
Miyamoto, 2009	Japan	No	NS	—	—	—	2Dfluo	Template	Open	C2-C7	29	Multiple	10
Mueller, 2010	Germany	NR	RCS	—	—	—	2Dfluo	FH	Open	C2	27	Trauma	11
Neo, 2005	Japan	No	NS	—	—	—	2Dfluo	FH	Open	C2-C6	18	Multiple	12
Niu, 2022	China	Yes	RCS	Non-nav vs template	—	—	2Dfluo	FH	Open	C1-C2	11	Trauma	16
								Template			12		
Park, 2021	South Korea	No	RCS	—	—	—	None	FH	Open	C3-C7	22	Multiple	10
Pham, 2018	USA	No	RCS	—	—	—	None	FH	Open	C2	24	Multiple	12
Pu, 2018	China	No	RCS	Non-nav vs template	—	—	2Dfluo	FH	Open	C1-C2	24	Trauma	18
								Template			25		
Scubbia, 2009	USA	No	PCS	—	—	—	None	FH	Open	C2	55	Multiple	12
Tofuku, 2012	Japan	No	RCS	—	—	—	2Dfluo	FH	Open	C2-C7	32	Trauma	11
Wang, 2013	China	No	NS	—	—	—	2Dfluo	FH	Open	C3-C7	214	Multiple	13
Wang, 2019	China	No	RCS	—	—	—	2Dfluo	Template	Open	C1-C2	19	Trauma	10
Wu, 2012	China	No	NS	—	—	—	2Dfluo	FH	Open	C2	10	Trauma	9
Wu, 2022	China	NR	RCS	Template vs template	—	—	2Dfluo	Template	Open	C2	44	Trauma	9
Yeom, 2008	South Korea	Yes	PCS	—	—	—	2Dfluo	FH	Open	C2	23	Multiple	13
Yoshii, 2016	Japan	No	PCS	Non-nav vs non-nav	—	—	iCT/2Dfluo	FH	Open	C2-C7	70	NS	12
Yukawa, 2009	Japan	NR	NS	—	—	—	2Dfluo	FH	Open	C2-C7	144	Trauma	12

Abbreviations: 2Dfluo, 2D fluoroscopy; CA, critical appraisal score according to the MINORS-criteria; CBCT, cone-beam computed tomography; CPS, cervical pedicle screws; DG, degenerative; FH, free-hand; iCT, intraoperative computed tomography; LMS, lateral mass screw; MIS, minimally invasive surgery; nav, navigated; non-nav, non-navigated; MT, medtronic; MX, mazor X; NR, not reported; NS, not specified; PCS, prospective cohort study; preop CT, preoperative computed tomography; RCS, retrospective cohort study; TPS, thoracic pedicle screws.

^a^Number of all patients included in the study, the number of patients specifically undergoing cervical pedicle screw insertion was not reported separately.

Overall, 37 studies assessed 4969 navigated screws reporting accuracy of placement rates of 79%-100% (e.g., screws placed completely in the pedicle or with a minor breach), 30 studies assessed 6603 non-navigated screws reporting rates of 67%-100%, and ten studies assessed 1104 screws placed using a 3D-printed guiding template reporting rates of 93%-100% (Supplement 3).

### Critical Appraisal and Publication Bias

The mean MINORS score for the 8 comparative studies was 17.5 (SD 2.4; range 15-20). For the 29 non-comparing studies assessing navigated screws, the mean MINORS score was 10.8 (SD 1.5; range 8-14), for the 22 studies assessing non-navigated screws it was 11.5 (SD 1.3; range 9-14), and for the ten studies assessing screws placed with a 3D-printed guiding template it was 10.6 (SD 1.3; range 9-13).

After critical appraisal, 41 studies were excluded from the primary analyses because they did not include a consecutive group of patients or no independent observer assessed the accuracy of placement of pedicle screws, and 8 studies because they only assessed screws placed with a patient-specific 3D-printed guiding template (Figure 1). Details on the critical appraisal for each study can be found in Supplement 4.

**Figure 2.**
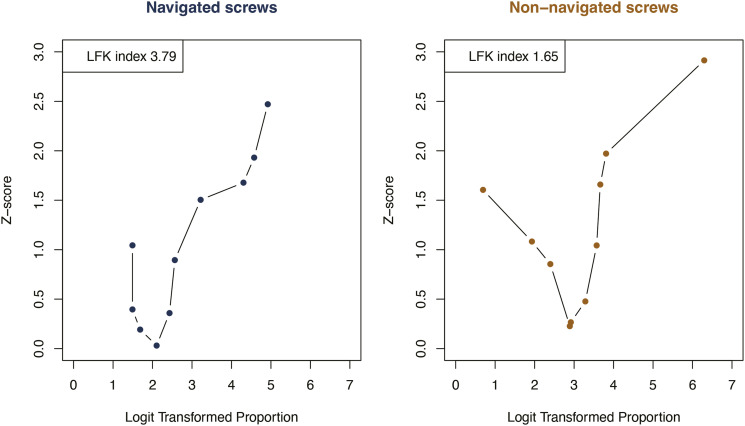
The Doi plots for the 18 studies included. Both Doi pots show asymmetry and indicate positive publication bias (an overestimated accuracy of placement for navigated and non-navigated screws).

The Doi plots for the 18 studies included in the primary analysis showed asymmetry for navigated screws (LFK index = 3.79), and for non-navigated screws (LFK index = 1.65), indicating positive publication bias (Figure 2).

### Screws Completely in the Pedicle or With a Minor Breach

**Figure 3.**
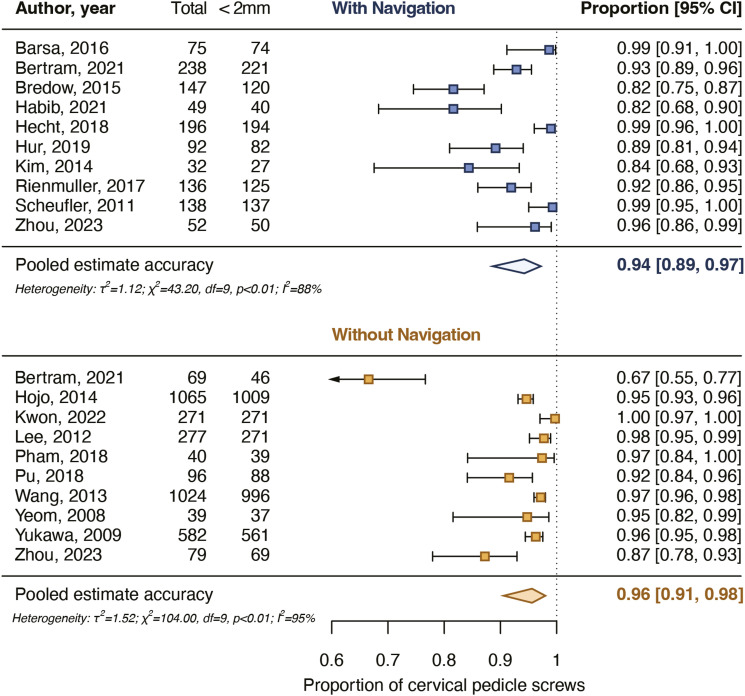
Pooled proportions for the accuracy of placement of cervical pedicle screws with a breach up to 2 mm. The pooled proportions did not differ significantly (*P* = .582) between navigated and non-navigated screws.

The exact number of patients included in the primary analysis could not be calculated because 2 studies did not report the number of patients undergoing cervical pedicle screw placement (1254-1415 patients) (Table 2). Ten studies assessed 1155 navigated screws of which 25%-53% were placed in C1-C2 (2 studies did not separately report the number of pedicle screws placed in C1-C2). Ten studies assessed 3542 non-navigated screws of which 12% were placed in C1-C2 (Supplement 5). The pooled proportion of navigated screws completely in the pedicle or with a minor breach was 94% [89%-97%], and the pooled proportion for non-navigated screws was 96% [91%-98%]. The pooled proportions did not differ significantly between the groups (*P* = .582) (Figure 3).

**Table 2.** Screw Details and Accuracy for All 18 Included Studies in the Primary Analysis.

Author, year	Patients	Levels	Classification used	Pedicle screw diameter (mm)	Screw insertion method	Total number of screws (% screws completely in the pedicle or with breach < 2 mm)		Number of screws completely in the pedicle (%)		Number of screws with a major breach > 4 mm (%)		Intraoperatively repositioned screws		Postoperatively revised screws	
						NAV	NON	NAV	NON	NAV	NON	NAV	NON	NAV	NON
Studies assessing navigated and non-navigated screw placement															
Bertram, 2021	157	C2-C7	2 mm cutoff	NR	FH	238 (93%)	69 (67%)	NR	NR	NR	NR	11 (5%)	0	0	0
Zhou, 2023	52	C1-C7	2 mm cutoff	3.5-4.0	FH/RB	52 (96%)	79 (87%)	43 (83%	48 (61%)	0	2 (3%)	0	3 (4%)	0	0
Studies assessing navigated screw placement															
Barsa, 2016	18	C5-C7	2 mm cutoff	NR	FH	75 (99%)	—	73 (97%)	—	1 (1%)	—	0	—	0	—
Bredow, 2015	64	C2-C7	2 mm cutoff	NR	FH	147 (82%)	—	67 (46%)	—	3 (2%)	—	NR	—	0	—
Habib, 2021	62	C3-C7	2 mm cutoff	3.5-4.0	FH	49 (82%)	—	32 (65%)	—	7 (14%)	—	1^a^	—	7^a^	—
Hecht, 2018	64^b^	C2-C7	2 mm cutoff	≥3.5	FH	196 (99%)	—	152 (78%)	—	0	—	5 (3%)	—	3^a^	—
Hur, 2019	48	C2	2 mm cutoff	NR	FH	92 (89%)	—	62 (67%)	—	4 (4%)	—	10 (11%)	—	0	—
Kim, 2014	18	C2	2 mm cutoff	NR	FH	32 (84%)	—	23 (72%)	—	NR	—	NR	—	0	—
Rienmuller, 2017	107^b^	C2-C7	2 mm cutoff	NR	FH	136 (92%)	—	97 (71%)	—	6 (4%)	—	NR	—	1 (1%)	—
Scheufler, 2011	27	C1-C7	2 mm cutoff	NR	FH	138 (99%)	—	117 (85%)	—	0	—	0	—	0	—
Studies assessing non-navigated screw placement															
Hojo, 2014	283	C2-C7	half screw diameter	3.5-4.0	FH	—	1065 (95%)	—	907 (85%)	—	NR	—	NR	—	3 (1%)
Kwon, 2022	57	C3-C7	half screw diameter	3.5	FH	—	271 (100%)	—	217 (80%)	—	0	—	NR	—	NR
Lee, 2012	50	C3-C7	half screw diameter	4.0	FH	—	277 (98%)	—	216 (78%)	—	NR	—	NR	—	0
Pham, 2018	24	C2	half screw diameter	3.5-4.0	FH	—	40 (98%)	—	33 (83%)	—	0	—	0	—	0
Pu, 2018^c^	24	C1-C2	2 mm cutoff	3.5	FH	—	96 (92%)	—	72 (75%)	—	0	—	NR	—	0
Wang, 2013	214	C3-C7	2 mm cutoff	3.5-4.0	FH	—	1024 (97%)	—	895 (87%)	—	NR	—	NR	—	2 (10%)
Yeom, 2008	23	C2	2 mm cutoff	3.5-4.0	FH	—	39 (95%)	—	31 (79%)	—	1 (3%)	—	0	—	0
Yukawa, 2009	144	C2-C7	half screw diameter	3.5-4.0	FH	—	582 (96%)	—	528 (91%)	—	NR	—	1 (1%)	—	5 (9%)

Abbreviations: FH, free-hand; NAV, navigated; NON, non-navigated; NR, not reported; RB, robotics.

^a^Numbers reported for all patients included in the study (including numbers for other screws than cervical pedicle screws).

^b^Number of all patients included in the study, the number of patients specifically undergoing cervical pedicle screw insertion was not reported separately.

^c^Cervical pedicle screws inserted using a 3D-printed guiding template were excluded.

### Screws Completely in the Pedicle

**Figure 4.**
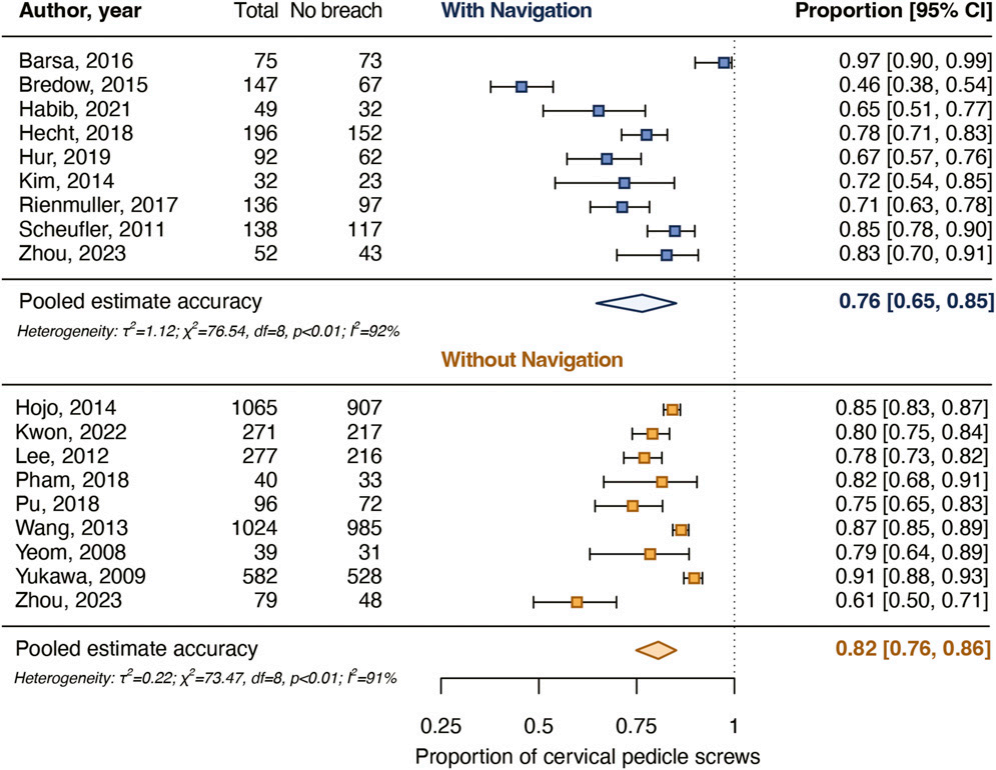
Pooled proportions for the accuracy of placement of cervical pedicle screws placed completely in the pedicle. The pooled proportions did not differ significantly (*P* = .359) between navigated and non-navigated screws.

Nine studies reported rates for screws placed completely in the pedicle varying from 46% to 97% for 885 navigated screws and nine studies of 61%-91% for 3473 non-navigated screws (Table 2). The pooled proportion of navigated screws completely in the pedicle was 76% [65%-85%] and for non-navigated screws it was 82% [76%-86%]. The pooled estimates did not differ significantly between the groups (*P* = .359) (Figure 4).

### Screws With a Major Breach

Eight studies reported rates for majorly breaching screws of 0%-14% for 885 navigated screws and five studies of 0%-3% for 525 non-navigated screws (Table 2). The pooled proportion of majorly breaching navigated screws was 1.4% [.4%-5.2%] and for severely deviating non-navigated screws it was .4% [.1%-3.4%]. The pooled estimates did not differ significantly between the groups (*P* = .357).

### Screws Repositioned Intraoperatively

**Figure 5.**
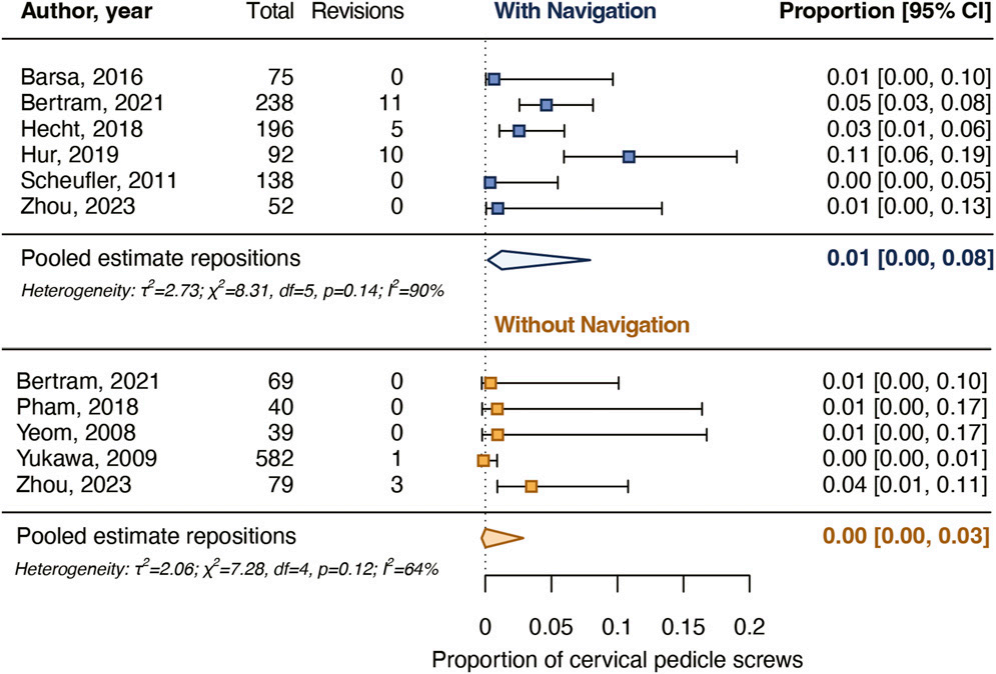
Pooled proportions for intraoperative cervical pedicle screw repositions. The pooled proportions did not differ significantly (*P* = 0.379) between navigated and non-navigated screws.

Six studies reported intraoperative screw reposition rates of 0%-11% for 791 navigated screws and five studies of 0%-4% for 809 non-navigated screws (Table 2). The pooled proportion for intraoperatively repositioned navigated screws was 1.3% [.2%-7.9%] and for non-navigated screws it was .3% [.0%-3.2%] (Figure 5). The pooled estimates did not differ significantly between the groups (*P* = .379).

### Screws Revised as a Result of Postoperative Imaging

**Figure 6.**
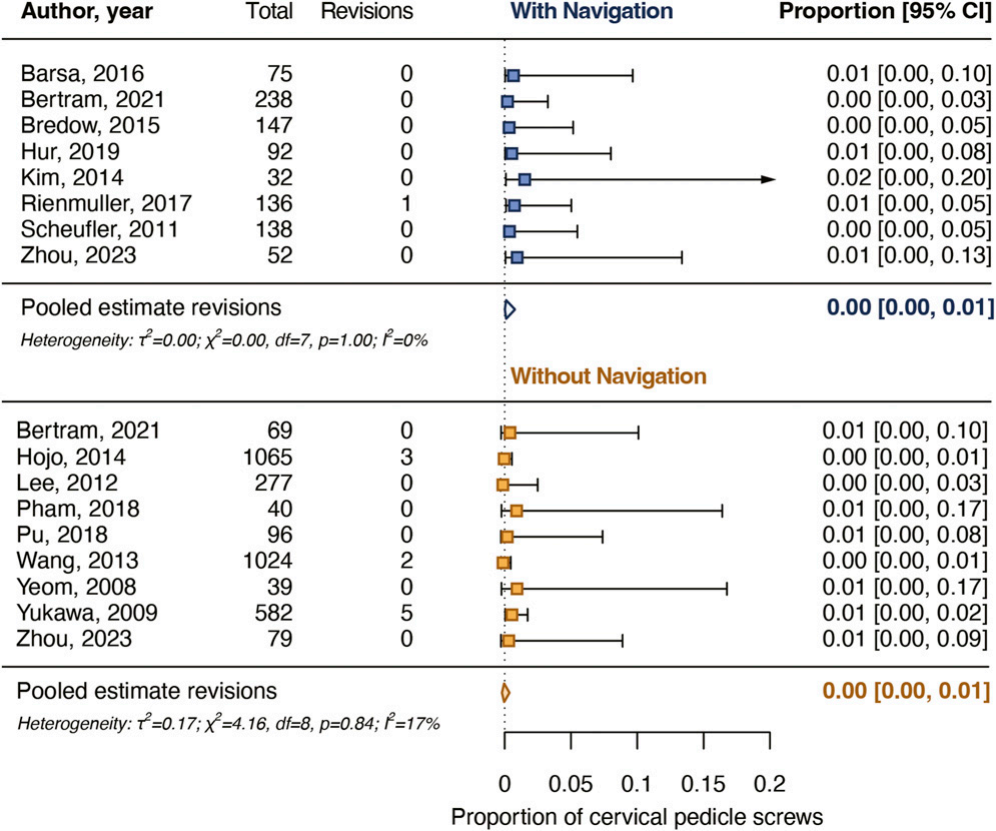
Pooled proportions for postoperative cervical pedicle screw revisions. The pooled proportions did not differ significantly (*P* = .398) between navigated and non-navigated screws.

The rates of screws revised as a result of postoperative imaging were reported in 8 studies for 910 navigated screws (0%-1%) and in 9 studies for 3271 non-navigated screws (0%-9%) (Table 2). The pooled proportion for screw revision as a result of postoperative imaging for navigated screws was .1% [.0%-.8%] and for non-navigated screws it was .3% [.1%-.7%] (Figure 6). The pooled estimates did not differ significantly between the groups (*P* = .398).

### Screws Placed With Patient-Specific 3D-Printed Guiding Templates

In 2 studies cervical pedicle screws were placed with a patient-specific 3D-printed template and an independent observer assessed the accuracy of placement of pedicle screws in a consecutive group of patients on CT. Both studies reported that 98% of the screws were placed completely in the pedicle (98 and 126 screws) and that 2% had a minor breach (both 2 screws) (Supplement 3).

## Discussion

This systematic review and meta-analysis assessed the accuracy of placement of cervical pedicle screws placed with the help of intraoperative navigation compared with screws placed without navigation. Eighteen non-randomized observational studies were included for analysis. The pooled accuracy of placement did not differ between navigated and non-navigated cervical pedicle screws, neither for screws placed completely in the pedicle nor for screws with a breach of <2 mm.

Multiple systematic reviews found that, with the help of navigation equipment, more pedicle screws were placed accurately in the thoracolumbar spine compared with screws placed without navigation.^8,9,90,91^ Some systematic reviews even included randomized controlled trials only.^90,91^ The current review did not find that the accuracy of placement for cervical pedicle screws increased if navigation equipment was used for screw placement. Most included studies were retrospective and non-comparative. Comparative studies allow for a more homogenous comparison of the screw placement accuracy, and may be less heterogenous regarding indications for surgery and patient characteristics. The current review identified 8 comparative studies, of which 7 reported that the accuracy of placement of cervical pedicle screw improved if surgeons used an intraoperative navigation system. However, we could only include 2 of the comparing studies in the meta-analysis, because only those 2 included a consecutive group of patients, and reported that an independent observer assessed the accuracy of placement. In addition, no comparative study used randomization and, except for 1,^81^ all were case-control studies. The 7 case-control studies compared patients treated with a recently acquired spinal navigation system to a historical group of patients that underwent cervical spinal fixation without navigation. Such studies are prone to publication and selection bias, and their results should be interpreted carefully. In particular, when information is lacking regarding how and by whom the screw placement accuracy was measured. For instance, reliable accuracy measurement of cervical pedicle screws on CT depends on proper scan acquisition and adequate reader training.^92^

The complex setup of a navigation system before it can be applied for cervical pedicle screw placement may partly explain why surgeons do not seem to place screws more accurately with intraoperative navigation. The cervical spine is highly mobile,^93^ and, when operating, the surgical working field is relatively small. Both the mobility of the cervical spine and the small surgical working field demand secure handling of navigational hardware such as the trackable reference frame. During navigation setup, the reference frame is attached to the patient and registered to the spine’s bony anatomy with intraoperative imaging. After the registration, the navigation system’s cameras utilize the attached reference frame to indirectly track the registered bony anatomy. The mobility of the cervical spine requires surgeons to register the reference frame just before they start using the navigation system, preferably after exposing the bony surface of the vertebrae, to minimize the risk of relative shifting of the frame to the vertebrae caused by surgical manipulation.^94^ Also, the reference frame must be attached as close to the target vertebrae as possible for optimal registration and navigation accuracy. When instrumenting on axial cervical levels, the position of the reference frame may be less of a problem because the frame can be fixated outside the surgical working field on the rigid Mayfield head holder. However, when operating on subaxial levels, the reference frame must remain within the surgical working field, attached to the spinous process of for example the C2, T1, or T2 vertebrae. The proximity of the reference frame during navigation forces surgeons to constantly pay attention to avoid unnecessary touching and moving of the frame. If the reference frame is accidentally bumped, the accuracy of the navigation system may degrade.^95^ In addition, after placement of every pedicle screw, surgeons should check if the navigation system is still accurately tracking the vertebrae. Every screw placement potentially causes relative movement between individual cervical vertebrae and, thus, a relative movement toward the reference frame.^93,96^ Therefore, surgeons must be familiar with the navigation system due to the complex setup of cervical navigation.^95,97^ A slight oversight can quickly degrade the navigation system’s accuracy and, consequently, the accurate placement of cervical pedicle screws.

Only focusing on radiologic placement accuracy to evaluate the use of navigation equipment for cervical pedicle screw placement may be too simplified. Outcomes related to the patient or surgical procedure are far more essential than radiologic outcome measures. Patients could also benefit from fewer screw-related postoperative complications or less impact of the surgery. Spine surgeons could also benefit if they could treat more complex cases safely, or achieve a shorter or safer learning curve for placing cervical pedicle screws. The present review did not find that using navigation for cervical pedicle screw placement resulted in fewer screw revisions as a result of postoperative imaging but did identify some opportunities where using navigation may be beneficial. Spinal navigation systems may facilitate minimally invasive (percutaneous) surgery in the cervical spine. In 7 studies, cervical pedicle screws were placed via a minimally invasive approach, and in all of these studies, navigation equipment was used during screw placement. Spinal navigation systems may allow spine surgeons to determine the entry point and trajectory for cervical pedicle screws more easily, even without widely exposing the anatomical landmarks and surrounding soft tissue, thus operating via a minimal approach. Furthermore, without navigation, the learning curve for accurate cervical pedicle screw placement is long, and accurate placement strongly depends on the surgeon’s experience.^98-100^ The learning curve for navigated cervical pedicle instrumentation may be relatively safer and shorter with appropriate training and familiarity with the navigation system.^18,30,36,42,48,59,60^ Nevertheless, a spinal navigation system is not a substitute for surgeon’s skills but rather an enhancement. Anatomical knowledge and competence regarding cervical pedicle screw placement remain essential to conduct the procedure safely. Surgeons cannot solely depend on a navigation system and must also be able to perform/end the surgery safely without navigation.

A cheaper alternative to intraoperative navigation for cervical pedicle screw placement is using pre-printed 3D templates for drilling or screwing, which was applied in ten studies. The pre-printed templates are patient-specific, and surgeons can place cervical pedicle screws accurately using these templates even when the anatomy is complex. However, the use of patient-specific templates can be time-consuming in terms of production and intraoperative positioning. More importantly, the opportunity to perform minimally invasive surgery is limited as current templates require close contact with the exposed bony surface.

### Limitations

Our review and meta-analysis must be interpreted in light of their strengths and limitations. First, this systematic review adhered to the PRISMA guidelines, and a study protocol was pre-registered to PROSPERO. Second, we used a broad search strategy focused on cervical pedicle screws, which was carefully developed to ensure that no relevant articles were missed, and after screening all articles, we found no new studies via other identification methods. Third, the large number of studies fitting our inclusion criteria allowed for meta-analyses of only the 18 studies with highest methodological quality. As a supplement, meta-analyses were performed including all 59 studies (excluding the 8 studies that used a patient-specific 3D-printed guiding template for screw placement). Our findings remained the same for screws placed with a breach up to 2 mm although the pooled proportion of screws placed completely in the pedicle was higher when intraoperative navigation was used (Supplement 6). The current meta-analyses may be limited due to the heterogeneity of the included studies. Included studies differed in study design, indications for surgery, experience of the surgeons, surgical approach, and the navigation system used. The 18 studies included in the meta-analyses did not allow for sub-analyses for potential confounders, such as minimally invasive surgery, if robotics were used to insert pedicle screws, and the cervical levels operated on. One study included in the meta-analyses applied robotics, and in 2 studies minimally invasive surgery was performed. For screws placed in the axial and subaxial spine separately, the accuracy of placement rates were added as a supplement (Supplement 5). In addition, the Doi plots indicated positive publication bias, thus an overestimated accuracy of placement for navigated and non-navigated screws. Lastly, clinically relevant outcomes such as postoperative complications, length of stay, blood loss, and operating time were too heterogeneous to compare between the included studies.

### Future Research

Future studies assessing intraoperative navigation for cervical pedicle screw placement should also focus on outcomes such as shortening the learning curve, reducing the complexity of surgical cases, and performing minimally invasive procedures. The years of experience as a spine surgeon and his/her familiarity with the navigation system should be taken into account as well.

## Conclusion

This systematic review and meta-analysis found that the use of spinal navigation systems does not significantly improve the accuracy of placement of cervical pedicle screws compared to screws placed without navigation. However, spinal navigation systems can facilitate interesting opportunities such as minimally invasive surgery. Future studies evaluating intraoperative navigation for cervical pedicle screw placement should focus on the learning curve, postoperative complications, and the complexity of surgical cases while using proper methodology to assess and report accuracy of placement.

## Supplemental Material

Supplemental Material - Accurate Placement and Revisions for Cervical Pedicle Screws Placed With or Without Navigation: A Systematic Review and Meta-Analysis Supplemental Material for Accurate Placement and Revisions for Cervical Pedicle Screws Placed With or Without Navigation: A Systematic Review and Meta-Analysis by B. J. J. Bindels, B. E. G. Dronkers, M. L. J. Smits, and J. J. Verlaan in Global Spine Journal.
