# Supplementary material for: Accurate Placement and Revisions for Cervical Pedicle Screws Placed With or Without Navigation: A Systematic Review and Meta-Analysis
Source: Global Spine J. 2023 Aug 19;14(3):1018–37. doi: 10.1177/21925682231196456 (PMC11192121; doi:10.1177/21925682231196456)
Supplement: Supplemental Material - Accurate Placement and Revisions for Cervical Pedicle Screws Placed With or Without Navigation: A Systematic Review and Meta-Analysis [file sj-pdf-1-gsj-10.1177_21925682231196456.pdf]

Supplementary data

Supplement 1. Search strategy

PubMed

#1 "Surgery, Computer-Assisted"[Mesh] OR "Fluoroscopy"[Mesh] OR "Cone-Beam Computed Tomography"[Mesh] OR "Tomography, Spiral Computed"[Mesh] OR "Robotic Surgical Procedures"[Mesh] OR computer-assisted[Title/Abstract] OR navigat*[Title/Abstract] OR image-guid*[Title/Abstract] OR CT-guid*[Title/Abstract] OR CT-based[Title/Abstract] OR X-ray[Title/Abstract] OR fluoroscop*[Title/Abstract] OR C-arm*[Title/Abstract] OR robotic*[Title/Abstract] OR conventional[Title/Abstract] OR conventionally[Title/Abstract] OR freehand[Title/Abstract] OR free-hand[Title/Abstract]

#2 "Cervical Vertebrae"[Mesh]
OR cervic*[Title/Abstract]

#3 "Pedicle Screws"[Mesh] OR "Bone Screws"[Mesh] OR "Spinal Fusion"[Mesh] OR "Fracture Fixation"[Mesh] OR screw*[Title/Abstract] OR fixation[Title/Abstract] OR fusion[Title/Abstract] OR spondylosyndesis[Title/Abstract] OR spondylodesis[Title/Abstract] OR stabiliz* [Title/Abstract]

#1 AND #2 AND #3

Embase

#1 'computer assisted surgery'/exp/mj OR ‘fluoroscopy'/exp OR ‘cone beam computed tomography'/exp OR ‘spiral computer assisted tomography'/exp OR ‘robot assisted surgery'/exp OR ‘computer-assisted’:ab,ti OR ‘navigat*’:ab,ti OR ‘image-guid*’:ab,ti OR ‘CT-guid*’:ab,ti OR ‘CT-based’:ab,ti OR ‘X-ray’:ab,ti OR ‘fluoroscop*’:ab,ti OR ‘C-arm*’:ab,ti OR ‘robotic*’:ab,ti OR ‘conventional’:ab,ti OR ‘conventionally’:ab,ti OR ‘freehand’:ab,ti OR ‘free-hand’:ab,ti

#2 'cervical vertebrae'/exp OR ‘cervic*’:ab,ti

#3 'pedicle screw'/exp OR ‘bone screw'/exp OR ‘spine fusion'/exp OR ‘fracture fixation'/exp OR ‘screw*’:ab,ti OR ‘fixation’:ab,ti OR ‘fusion’:ab,ti OR ‘spondylosyndesis’:ab,ti OR ‘spondylodesis’:ab,ti OR ‘stabiliz* ’:ab,ti

#1 AND #2 AND #3

Cochrane

#1 MeSH descriptor: [Surgery, Computer-Assisted] explode all trees
#2 MeSH descriptor: [Fluoroscopy] explode all trees
#3 MeSH descriptor: [Cone-Beam Computed Tomography] explode all trees
#4 MeSH descriptor: [Tomography, Spiral Computed] explode all trees
#5 MeSH descriptor: [Robotic Surgical Procedures] explode all trees
#6 (computer-assisted):ti,ab,kw
#7 (navigat*):ti,ab,kw
#8 (image-guid*):ti,ab,kw

#9 (CT-guid*):ti,ab,kw

#10 (CT-based):ti,ab,kw

#11 (X-ray):ti,ab,kw

#12 (fluoroscop*):ti,ab,kw

#13 (c-arm*):ti,ab,kw

#14 (robotic*):ti,ab,kw

#15 (conventional):ti,ab,kw

#16 (conventionally):ti,ab,kw

#17 (freehand):ti,ab,kw

#18 (free-hand):ti,ab,kw

#19 #1 OR #2 OR # 9 OR #4 OR #5 OR #6 OR #7 OR #8 OR #9 OR #10 OR #11 OR #12 OR #13 OR #14 OR #15 OR #16 OR #17 OR #18

#20 MeSH descriptor: [Cervical Vertebrae] explode all trees

#21 (cervic*):ti,ab,kw

#22 #20 OR #21

#23 MeSH descriptor: [Pedicle Screws] explode all trees

#24 MeSH descriptor: [Bone Screws] explode all trees

#25 MeSH descriptor: [Spinal Fusion] explode all trees

#26 MeSH descriptor: [Fracture Fixation] explode all trees

#27 (screw*):ti,ab,kw

#28 (fixation):ti,ab,kw

#29 (fusion):ti,ab,kw

#30 (spondylosyndesis):ti,ab,kw

#31 (spondylodesis):ti,ab,kw

#32 (stabiliz*):ti,ab,kw

#33 #23 OR #24 OR #25 OR #26 OR #27 OR #28 OR #29 OR #30 OR #31 OR #32

#34 #19 AND #22 AND #33

Supplement 2. MINORS checklist adapted to our primary study outcome

---

1. A clearly stated aim: the question addressed should be precise and relevant in the light of available literature
0 – not reported
1 – reported but inadequate -> accuracy of placement was a secondary outcome
2 – reported and adequate -> accuracy of placement was the primary outcome

2. Inclusion of consecutive patients: all patients potentially fit for inclusion (satisfying the criteria for inclusion) have been included in the study during the study period (no exclusion or details about the reasons for exclusion)
0 – not reported
1 – reported but inadequate -> unclear if consecutive group of patients was included
2 – reported and adequate -> consecutive group of patients included during study period

3. Prospective collection of data: data were collected according to a protocol established before the beginning of the study
0 – not reported
1 – reported but inadequate -> retrospective study
2 – reported and adequate -> prospective study

4. Endpoints appropriate to the aim of the study: unambiguous explanation of the criteria used to evaluate the main outcome which should be in accordance with the question addressed by the study. Also, the endpoints should be assessed on an intention-to-treat basis.
0 – not reported
1 – reported but inadequate -> accuracy assessed on other image than CT or CBCT
2 – reported and adequate -> accuracy assessed on intraoperative or postoperative CT or CBCT

5. Unbiased assessment of the study endpoint: blind evaluation of objective endpoints and double-blind evaluation of subjective endpoints. Otherwise the reasons for not blinding should be stated
0 – not reported-> unclear who assessed the accuracy of placement
1 – reported but inadequate -> someone involved in the surgery assessed the accuracy of placement
2 – reported and adequate -> accuracy of placement was assessed by at least one independent observer

6. Follow-up period appropriate to the aim of the study: the follow-up should be sufficiently long to allow the assessment of the main endpoint and possible adverse events
0 – not reported
1 – reported but inadequate -> postoperative CT or CBCT without time-interval
2 – reported and adequate -> intraoperative or postoperative CT or CBCT with time-interval mentioned

7. Loss to follow up less than 5%: all patients should be included in the follow up. Otherwise, the proportion lost to follow up should not exceed the proportion experiencing the major endpoint
0 – not reported
1 – reported but inadequate -> not all patients underwent imaging on which screw accuracy of placement was assessed

2 – reported and adequate -> all patients who underwent cervical pedicle screw fixation received adequate imaging (CT or CBCT) on which screw accuracy of placement was assessed

8. Prospective calculation of the study size: information of the size of detectable difference of interest with a calculation of 95% confidence interval, according to the expected incidence of the outcome event, and information about the level for statistical significance and estimates of power when comparing the outcomes
0 – not reported
1 – reported but inadequate
2 – reported and adequate

<u>Additional criteria in the case of comparative study*</u>

9. An adequate control group: having a gold standard diagnostic test or therapeutic intervention recognized as the optimal intervention according to the available published data
0 – not reported
1 – reported but inadequate
2 – reported and adequate

10. Contemporary groups: control and studied group should be managed during the same time period (no historical comparison)
0 – not reported
1 – reported but inadequate -> case-control studies
2 – reported and adequate -> observational studies

11. Baseline equivalence of groups: the groups should be similar regarding the criteria other than the studied endpoints. Absence of confounding factors that could bias the interpretation of the results
0 – not reported
1 – reported but inadequate
2 – reported and adequate

12. Adequate statistical analyses: whether the statistics were in accordance with the type of study with calculation of confidence intervals or relative risk
0 – not reported
1 – reported but inadequate
2 – reported and adequate

*Only studies that directly compared navigated cervical pedicle screw placement to screw placement without navigation were critically appraised as comparative studies.

Supplement 3. Screw details and accuracy for all 67 included studies

Author, year	Patients	Levels	Classification used	Pedicle screw diameter (mm)	Screw insertion method	Total number of screws (% screws completely in the pedicle or with breach < 2mm)		Number of screws completely in the pedicle (%)		Number of screws with a major breach > 4mm (%)		Intraoperatively repositioned screws		Postoperatively revised screws	
						NAV	NON	NAV	NON	NAV	NON	NAV	NON	NAV	NON
**Studies assessing navigated and non-navigated screw placement**															
Bertram, 2021	157	C2-C7	2mm cutoff	NR	FH	238 (93%)	69 (67%)	NR	NR	NR	NR	11 (5%)	0	0	0
Harel, 2019	14	C2	2mm cutoff	NR	FH	10 (100%)	9 (89%)	8 (80%)	5 (50%)	0	0	1 (10%)	0	0	0
Inoue, 2022	37*	C7	2mm cutoff	NR	FH	24 (100%)	10 (80%)	24 (100%)	4 (40%)	0	1 (10%)	NR	NR	0	0
Lee J, 2020	34	C2	2mm cutoff	NR	FH	32 (97%)	26 (96%)	30 (94%)	23 (88%)	0	1 (4%)	1 (3%)	0	0	0
Su, 2022	58	C1-C4	2mm cutoff	NR	FH/RB	180 (97%)	194 (91%)	163 (91%)	138 (71%)	1 (1%)	2 (1%)	NR	NR	0	0
Takamatsu, 2022	11	C2-C7	2mm cutoff	NR	FH	26 (100%)	16 (81%)	25 (96%)	14 (88%)	0	0	2 (8%)	0	0	0
Tanaka, 2020	25	C2-C7	2mm cutoff	NR	FH	96 (100%)	34 (82%)	81 (84%)	17 (50%)	0	0	NR	NR	0	0
Zhou, 2023	52	C1-C7	2mm cutoff	3.5-4.0	FH/RB	52 (96%)	79 (87%)	43 (83%	48 (61%)	0	2 (3%)	0	3 (4%)	0	0
**Studies assessing navigated screw placement**															
Barsa, 2016	18	C5-C7	2mm cutoff	NR	FH	75 (99%)	-	73 (97%)	-	1 (1%)	-	0	-	0	-
Bohoun, 2019	12*	NS	2mm cutoff	NR	FH	77 (100%)	-	77 (100%)	-	0	-	NR	-	0	-
Bredow, 2015	64	C1-C7	2mm cutoff	NR	FH	147 (82%)	-	67 (46%)	-	3 (2%)	-	NR	-	0	-
Carl, 2019	16	C2	2mm cutoff	3.5	FH	26 (96%)	-	23 (88%)	-	0	-	0	-	0	-
Chachan, 2018	44	C2-C7	2mm cutoff	NR	FH	241 (97%)	-	224 (93%)	-	0	-	NR	-	0	-
Gan, 2018	82	C2-C7	2mm cutoff	NR	FH	297 (94%)	-	229 (77%)	-	4 (1%)	-	0	-	6 (2%)	-
Habib, 2021	62	C3-C7	2mm cutoff	3.5-4.0	FH	49 (82%)	-	32 (65%)	-	7 (14%)	-	1**	-	7**	-
Hecht, 2018	64*	C2-C7	2mm cutoff	≥3.5	FH	196 (99%)	-	152 (78%)	-	0	-	5 (3%)	-	3**	-
Hur, 2019	48	C2	2mm cutoff	NR	FH	92 (89%)	-	62 (67%)	-	4 (4%)	-	10 (11%)	-	0	-
Ito, 2008	50*	C1-C7	2mm cutoff	≥3.5	FH	176 (100%)	-	171 (97%)	-	0	-	NR	-	0	-
Kim, 2014	18	C2	2mm cutoff	NR	FH	32 (84%)	-	23 (72%)	-	NR	-	NR	-	0	-
Kisinde, 2022	12	C2-C7	2mm cutoff	3.5	RB	88 (100%)	-	74 (84%)	-	0	-	0	-	0	-
Komatsubara, 2017	56	C2-C7	2mm cutoff	NR	FH	185 (94%)	-	NR	-	NR	-	NR	-	NR	-
Kumar, 2019	219*	C2-C7	2mm cutoff	NR	FH	241 (97%)	-	226 (94%)	-	0	-	8 (3%)	-	0	-
Lang, 2016	20	C2-C3	2mm cutoff	NR	FH	80 (100%)	-	70 (88%)	-	0	-	NR	-	0	-
Oikonomidis, 2020	28	C3-C7	2mm cutoff	NR	FH	87 (79%)	-	32 (37%)	-	6 (7%)	-	NR	-	NR	-
Rajan, 2010	18	NS	2mm cutoff	NR	FH	98 (98%)	-	89 (91%)	-	0	-	NR	-	NR	-
Rienmuller, 2017	107*	C2-C7	2mm cutoff	NR	FH	136 (92%)	-	97 (71%)	-	6 (4%)	-	NR	-	1 (1%)	-
Satake, 2021	47	C3-C6	2mm cutoff	NR	FH	207 (98%)	-	183 (88%)	-	0	-	NR	-	0	-
Scheufler, 2011	27	C1-C7	2mm cutoff	NR	FH	138 (99%)	-	117 (85%)	-	0	-	0	-	0	-
Shimokawa, 2017	128*	C2-C7	2mm cutoff	NR	FH	648 (98%)	-	530 (82%)	-	3 (1%)	-	NR	-	0	-
Shin, 2022	51	C2-C7	2mm cutoff	NR	FH	156 (99%)	-	146 (94%)	-	0	-	5 (3%)	-	0	-
Sugimoto, 2010	17	C2-C7	2mm cutoff	NR	FH	76 (100%)	-	74 (97%)	-	NR	-	NR	-	NR	-
Sugimoto, 2017	18	C2-C7	2mm cutoff	NR	FH	83 (86%)	-	NR	-	NR	-	NR	-	NR	-
Tauchi, 2013	46	C2-C7	2mm cutoff	NR	FH	196 (88%)	-	150 (77%)	-	15 (8%)	-	0	-	0	-
Tian, 2012	14	C2-C3	half screw diameter	3.5-4.0	FH	51 (100%)	-	NR	-	0	-	0	-	0	-
Tokioka, 2019	86	C2-C6	2mm cutoff	4.0	FH	294 (96%)	-	NR	-	NR	-	NR	-	0	-
Wada, 2020	64	C2-C7	2mm cutoff	NR	Template	317 (100%)	-	305 (96%)	-	0	-	NR	-	0	-
Zausinger, 2009	12	C1-C2	2mm cutoff	NR	FH	24 (92%)	-	19 (79%)	-	0	-	1 (4%)	-	0	-

Zhang, 2022	22	C3-C7	2mm cutoff	NR	FH	118 (93%)	-	83 (70%)	-	0	-	NR	-	0	-
**Studies assessing non-navigated screw placement**															
Cao, 2017	87	C1-C2	2mm cutoff	3.5-4.0	FH	-	306 (100%)	-	264 (86%)	-	0	-	NR	-	0
Farshad, 2022	12	C2-C7	2mm cutoff	NR	Template	-	86 (100%)	-	82 (95%)	-	0	-	NR	-	0
Hey, 2020	21	C3-C7	2mm cutoff	4.0	FH	-	107 (97%)	-	74 (69%)	-	1 (1%)	-	NR	-	0
Hojo, 2014	283	C2-C7	half screw diameter	3.5-4.0	FH	-	1065 (95%)	-	907 (85%)	-	NR	-	NR	-	3 (1%)
Jiang, 2016	32	C1-C2	2mm cutoff	3.5	Template	-	128 (100%)	-	126 (98%)	-	0	-	0	-	0
Kaneyama, 2015	20	C3-C6	2mm cutoff	3.5	Template	-	80 (100%)	-	78 (98%)	-	0	-	NR	-	0
Kwon, 2022	57	C3-C7	half screw diameter	3.5	FH	-	271 (100%)	-	217 (80%)	-	0	-	NR	-	NR
Lee B, 2020	25	C1	2mm cutoff	3.5	FH	-	49 (90%)	-	31 (63%)	-	3 (6%)	-	NR	-	0
Lee, 2012	50	C3-C7	half screw diameter	4.0	FH	-	277 (98%)	-	216 (78%)	-	NR	-	NR	-	0
Li, 2022	32	C3-C7	2mm cutoff	NR	FH	-	694 (96%)	-	NR	-	NR	-	NR	-	NR
Liu, 2020	32	C3-C7	2mm cutoff	3.5	FH	-	257 (97%)	-	231 (90%)	-	0	-	NR	-	0
Lu, 2009	25	C2-C7	2mm cutoff	≥3.0	Template	-	88 (97%)	-	14 (16%)	-	0	-	0	-	0
Mahesh, 2020	99	C3-C7	half screw diameter	3.5	FH	-	577 (97%)	-	429 (74%)	-	NR	-	NR	-	0
Miyamoto, 2009	29	C2-C7	half screw diameter	3.5	Template	-	103 (93%)	-	NR	-	2 (2%)	-	NR	-	0
Mueller, 2010	27	C2	2mm cutoff	4.0	FH	-	47 (83%)	-	26 (55%)	-	3 (6%)	-	NR	-	0
Neo, 2005	18	C2-C6	2mm cutoff	4.0	FH	-	86 (85%)	-	61 (71%)	-	7 (8%)	-	NR	-	0
Niu, 2022	11	C1-C2	2mm cutoff	NR	FH	-	42 (81%)	-	NR	-	NR	-	NR	-	0
	12				Template		46 (96%)		NR		NR		NR		0
Park, 2021	22	C3-C7	half screw diameter	3.5-4.0	FH	-	78 (92%)	-	62 (79%)	-	NR	-	NR	-	0
Pham, 2018	24	C2	half screw diameter	3.5-4.0	FH	-	40 (98%)	-	33 (83%)	-	0	-	0	-	0
Pu, 2018	24	C1-C2	2mm cutoff	3.5	FH	-	96 (92%)	-	72 (75%)	-	0	-	NR	-	0
	25				Template		100 (100%)		98 (98%)		0		NR		0
Scubbia, 2009	55	C2	half screw diameter	3.5	FH	-	100 (98%)	-	85 (85%)	-	1 (1%)	-	0	-	0
Tofuku, 2012	32	C2-C7	half screw diameter	3.5	FH	-	127 (96%)	-	112 (88%)	-	NR	-	NR	-	NR
Wang, 2013	214	C3-C7	2mm cutoff	3.5-4.0	FH	-	1024 (97%)	-	895 (87%)	-	NR	-	NR	-	2 (10%)
Wang, 2019	19	C1-C2	2mm cutoff	NR	Template	-	68 (97%)	-	64 (94%)	-	NR	-	NR	-	0
Wu, 2012	10	C2	2mm cutoff	3.5	FH	-	20 (100%)	-	17 (85%)	-	0	-	NR	-	0
Wu, 2022	44	C2	2mm cutoff	3.5	Template	-	88 (98%)	-	77 (88%)	-	NR	-	NR	-	NR
Yeom, 2008	23	C2	2mm cutoff	3.5-4.0	FH	-	39 (95%)	-	31 (79%)	-	1 (3%)	-	0	-	0
Yoshii, 2016	70	C2-C7	half screw diameter	3.5-4.0	FH	-	282 (96%)	-	251 (89%)	-	NR	-	NR	-	1 (0.4%)
Yukawa, 2009	144	C2-C7	half screw diameter	3.5-4.0	FH	-	582 (96%)	-	528 (91%)	-	NR	-	1 (1%)	-	5 (9%)

**Number of all patients included in the study, the number of patients specifically undergoing cervical pedicle screw insertion was not reported separately, **Numbers reported for all patients included in the study (including numbers for other screws than cervical pedicle screws). Abbreviations: FH = free-hand, NAV = navigated, NON = non-navigated, NR = not reported, RB = robotic*

Supplement 4. . The Methodological Index for Non-Randomized Studies (MINORS) criteria for all 67 included studies

Author, year	1. Aim	2. Consecutive patient	3. Prospective data	4. Endpoints appropriate	5. Unbiased assessment	6. Follow-up period appropriate	7. <5% loss to follow-up	8. Prospective calculation	9. Control group	10. Contemporary group	11. Baseline equivalence	12. Adequate statistics	Score
**Studies assessing navigated and non-navigated screw placement**													
Bertram, 2021	2	2	2	2	2	1	2	0	2	1	2	2	20
Harel, 2019	2	1	1	2	0	1	2	0	2	1	2	2	16
Inoue, 2022	2	1	1	2	0	1	1	0	2	1	2	2	15
Lee J, 2020	2	1	1	2	2	2	2	0	2	1	2	2	19
Su, 2022	2	2	2	2	0	2	2	0	2	2	2	2	20
Takamatsu, 2022	1	2	1	2	0	1	2	0	1	1	2	2	15
Tanaka, 2021	2	1	1	2	0	2	2	0	1	1	1	2	15
Zhou, 2023	2	2	1	2	2	2	2	1	1	1	2	2	20
**Studies assessing navigated screw placement**													
Barsa, 2016	2	2	2	2	2	2	2	0					14
Bohoun, 2019	2	2	1	2	0	1	2	0					10
Bredow, 2015	2	2	1	2	2	2	1	0					12
Carl, 2019	2	2	1	2	0	2	2	0					11
Chachan, 2018	2	2	2	2	0	2	2	0					12
Gan, 2021	2	2	1	2	0	2	2	0					11
Habib, 2021	2	2	1	2	2	1	1	0					11
Hecht, 2018	2	2	2	2	2	1	2	0					13
Hur, 2019	2	2	1	2	2	1	2	0					12
Ito, 2008	2	2	2	2	0	1	2	0					11
Kim, 2014	2	2	1	2	2	1	1	0					11
Kisinde, 2022	2	2	1	2	1	1	1	0					10
Komatsubara, 2017	2	2	1	2	0	1	2	0					10
Kumar, 2019	2	1	1	2	0	2	2	0					10
Lang, 2016	2	1	1	2	0	1	2	0					9
Oikonomidis, 2020	2	1	1	2	2	1	1	0					10
Rajan, 2010	2	1	2	2	2	1	2	0					12
Rienmuller, 2017	2	2	2	2	2	2	2	0					14
Satake, 2021	2	2	1	2	0	2	2	0					11
Scheufler, 2011	2	2	1	2	2	2	2	0					13
Shimokawa, 2017	2	1	1	2	0	2	2	0					10
Shin, 2022	2	1	1	2	0	1	1	0					8
Sugimoto, 2010	1	1	1	2	0	2	2	0					9
Sugimoto, 2017	2	2	1	2	0	1	2	0					10
Tauchi, 2012	2	2	1	2	0	1	2	0					10
Tian, 2012	2	2	1	2	0	2	2	0					11
Tokioka, 2019	1	2	1	2	0	1	2	0					9
Wada, 2020	2	1	1	2	0	1	2	0					9
Zausinger, 2009	2	1	1	2	0	2	2	0					10
Zhang, 2022	2	2	1	2	0	1	2	0					10
**Studies assessing non-navigated screw placement**													

Cao, 2017	1	2	1	2	0	2	2	0	10
Farshad, 2022	2	1	1	2	2	2	1	0	11
Hey, 2020	2	2	2	2	1	1	2	0	12
Hojo, 2014	2	2	1	2	2	2	1	0	12
Jiang, 2016	2	2	2	2	2	1	2	0	13
Kaneyama, 2015	2	2	1	2	0	1	2	0	10
Kwon, 2022	2	2	2	2	2	1	2	0	13
Lee B, 2020	2	2	1	2	0	2	2	0	11
Lee, 2012	2	2	1	2	2	1	2	0	12
Li, 2022	2	1	1	2	0	1	2	0	9
Liu, 2020	2	2	2	2	1	1	2	2	14
Lu, 2009	2	1	2	2	2	1	2	0	12
Mahesh, 2020	2	2	1	2	1	1	2	0	11
Miyamoto, 2009	2	2	1	2	0	1	2	0	10
Mueller, 2010	2	2	1	2	0	2	2	0	11
Neo, 2005	2	2	1	2	1	2	2	0	12
Niu, 2022	2	1	1	2	0	2	2	0	10
Park, 2021	2	2	1	2	0	1	2	0	10
Pham, 2018	2	2	1	2	1	2	2	0	12
Pu, 2018	2	2	1	2	2	1	2	0	12
Scubbia, 2009	2	2	2	2	0	2	2	0	12
Tofuku, 2012	2	2	1	2	0	2	2	0	11
Wang, 2013	2	2	1	2	2	2	2	0	13
Wang, 2019	2	2	1	2	0	1	2	0	10
Wu, 2012	1	1	1	2	0	2	2	0	9
Wu, 2022	2	1	1	2	1	1	1	0	9
Yeom, 2008	2	2	2	2	2	1	2	0	13
Yoshii, 2016	2	2	2	2	0	2	2	0	12
Yukawa, 2009	2	2	1	2	2	1	2	0	12

Items are scored 0 if the item is not reported, 1 if inadequately reported, or 2 if adequately reported. Comparative studies can score a maximum of 24 points, and non-comparative studies can score 16 points.

Supplement 5. Accuracy of placement for the axial spine (C1-C2) and subaxial cervical spine (C3-C7) of the 18 included studies in the meta-analyses.

Axial spine (C1-C2)

Eight studies placed navigated pedicle screws in C1-C2, and six reported the accuracy of placement separately. The six studies placed 287 pedicle screws and reported 84-100% of the screws to be completely in the pedicle or with a minor breach. One study did not separately report the number of screws completely in the pedicle. For the remaining 241 screws, 63-96% were reported to be completely in the pedicle.

Seven studies placed non-navigated pedicle screws in C1-C2, and all reported the accuracy of placement separately. In total, 439 pedicle screws were placed, of which 89-98% were reported to be completely in the pedicle or having a minor breach. One study did not separately report the number of screws placed completely in the pedicle. For the remaining 417 pedicle screws, 62-83% were reported to be completely in the pedicle.

Subaxial cervical spine (C3-C7)

Eight studies placed navigated pedicle screws in C3-C7, and six reported the accuracy of placement separately. The six studies placed 536 pedicle screws and reported 78-100% of the screws to be completely in the pedicle or with a minor breach. One study did not separately report the number of screws completely in the pedicle. For the remaining 344 screws, 42-97% were reported to be completely in the pedicle.

Seven studies placed non-navigated pedicle screws in C3-C7, and all reported the accuracy of placement separately. In total, 3103 pedicle screws were placed, of which 55-100% were reported to be completely in the pedicle or having a minor breach. One study did not separately report the number of screws placed completely in the pedicle. For the remaining 3056 pedicle screws, 50-88% were reported to be completely in the pedicle.

Table for Supplement 5. Accuracy of placement for the axial spine (C1-C2) and subaxial cervical spine (C3-C7) of the 18 included studies in the meta-analyses.

				Axial spine (C1-C2)				Subaxial spine (C3-C7)			
Author, year	Levels	Pedicle screw diameter (mm)	Screw insertion method	Total number of screws placed (% screws completely in the pedicle or with breach < 2mm)		Number of screws placed completely in the pedicle (%)		Total number of screws (% screws completely in the pedicle or with breach < 2mm)		Number of screws placed completely in the pedicle (%)	
				NAV	NON	NAV	NON	NAV	NON	NAV	NON
**Studies assessing navigated and non-navigated screw placement**											
Bertram, 2021	C2-C7	NR	FH	46 (96%)	22 (91%)	NR	NR	192 (87%)	47 (55%)	NR	NR
Zhou, 2023	C1-C7	3.5-4.0	FH/RB	42 (95%)	71 (89%)	35 (83%)	44 (62%)	10 (100%)	8 (75%)	8 (80%)	4 (50%)
**Studies assessing navigated screw placement**											
Barsa, 2016	C5-C7	NR	FH	-	-	-	-	75 (99%)	-	73 (97%)	-
Bredow, 2015	C2-C7	NR	FH	27 (96%)	-	17 (63%)	-	120 (78%)	-	50 (42%)	-
Habib, 2021	C3-C7	3.5-4.0	FH	-	-	-	-	49 (82%)	-	32 (65%)	-
Hecht, 2018	C2-C7	≥3.5	FH	NR	-	NR	-	NR	-	NR	-
Hur, 2019	C2	NR	FH	92 (89%)	-	62 (67%)	-	-	-	-	-
Kim, 2014	C2	NR	FH	32 (84%)	-	23 (72%)	-	-	-	-	-
Rienmuller, 2017	C2-C7	NR	FH	NR	-	NR	-	NR	-	NR	-
Scheufler, 2011	C1-C7	NR	FH	48 (100%)	-	46 (96%)	-	90 (99%)	-	71 (79%)	-
**Studies assessing non-navigated screw placement**											
Hojo, 2014	C2-C7	3.5-4.0	FH	-	148 (89%)	-	114 (77%)	-	917 (96%)	-	793 (86%)
Kwon, 2022	C3-C7	3.5	FH	-	-	-	-	-	271 (100%)	-	217 (80%)
Lee, 2012	C3-C7	4.0	FH	-	-	-	-	-	277 (98%)	-	216 (78%)
Pham, 2018	C2	3.5-4.0	FH	-	40 (98%)	-	33 (83%)	-	-	-	-
Pu, 2018*	C1-C2	3.5	FH	-	96 (92%)	-	72 (75%)	-	-	-	-
Wang, 2013	C3-C7	3.5-4.0	FH	-	-	-	-	-	1024 (97%)	-	895 (87%)
Yeom, 2008	C2	3.5-4.0	FH	-	39 (95%)	-	31 (79%)	-	-	-	-
Yukawa, 2009	C2-C7	3.5-4.0	FH	-	23 (91%)	-	17 (74%)	-	559 (97%)	-	490 (88%)

** Cervical pedicle screws inserted using a 3D-printed guiding template were excluded. Abbreviations: FH = free-hand, NAV = navigated, NON = non-navigated, NR = not reported, RB = robotics*

Supplement 6. Meta-analyses for all 59 studies assessing navigated and non-navigated cervical pedicle screw placement.

Pooled proportions of cervical pedicle screws placed completely in the pedicle or with a minor breach:

Navigated screws: 97% [95% CI: 95% - 98%]

Non-navigated screws: 95% [9% CI: 93% - 97%]

The pooled proportions did not differ significantly between navigated and non-navigated screws (p = 0.155).

Pooled proportions of cervical pedicle screws placed completely in the pedicle:

Navigated screws: 86% [95% CI: 81% - 91%]

Non-navigated screws: 97% [9% CI: 74% - 83%]

The pooled proportion was significantly higher for navigated screws (p = 0.023).
